# Green Transformational Leadership and Employees’ Taking Charge Behavior: The Mediating Role of Personal Initiative and the Moderating Role of Green Organizational Identity

**DOI:** 10.3390/ijerph19074172

**Published:** 2022-03-31

**Authors:** Yuechao Du, Minghao Yan

**Affiliations:** 1 School of Management, Zhejiang University, Hangzhou 310058, China; ycdu@zju.edu.cn; 2 Yantai Institute, China Agricultural University, Yantai 264670, China

**Keywords:** green transformational leadership, personal initiative, taking charge behavior, green organizational identity

## Abstract

The green transformation of organizations requires employees not only to achieve environmentally friendly workflows within their duties, but also to go beyond their own current work and take personal initiative to realize the organizational goals. Employees’ taking charge behavior is a type of extra-role behavior that influences organizational change through constructive efforts. How can leaders increase employees’ environmental responsibility and efficiently promote their taking charge behaviors to help organizations make green changes? Based on self-determination theory and related research on green transformational leadership, this study explores the mechanisms and boundary conditions of how green transformational leadership influences employees’ taking charge behavior. Data were obtained through two-stage questionnaire surveys from 429 employees in Chinese manufacturing enterprises. The results show that green transformational leadership has a significantly positive impact on employees’ taking charge behavior and that personal initiative plays a mediating role. Furthermore, green organizational identity moderates the positive influence of green transformational leadership on employees’ personal initiative, and consequently, their taking charge behavior. These findings have theoretical implications for the green transformational leadership literature and managerial implications for practitioners.

## 1. Introduction

With the globalization of the economy, environmental issues are becoming increasingly prominent on a global scale. In recent years, as carbon neutrality and carbon peaking targets continue to be promoted, an increasing number of companies are becoming aware of the importance of environmental responsibility. These companies are not only striving for energy saving and emission reduction in their production by setting green targets to constrain manufacturing activities, but also seeking green change in their management models to reduce potential environmental hazards. In the practice of green organizational change, leaders play a key and central role [1]. There have been several types of leadership concepts for the study of green management, including sustainable leadership and green transformational leadership. Sustainable leadership is a leadership style that takes into consideration a comprehensive scope of complex interconnections among corporate profits, business growth, preservation of the environment, and social values, which emphasizes obtaining success in the long term based on strategic decision-making value [2]. Compared with sustainable leadership that has a broader connotation, green transformational leadership, which reflects the green values of leaders and explains the mechanisms by which leaders influence green organizational change, places more emphasis on the environmental benefits of the organization [3,4]. Green transformational leadership, which is also called environmental transformational leadership, is a leadership style that aims to motivate employees to achieve green goals, shape the green vision of employees and promote green change in the organization [5]. Previous research on green transformational leadership has illustrated its profound impact on employees’ environmental responsibility [6], organizational citizenship behavior [7], organizational citizenship behavior for the environment [8,9], and its importance for sustainable development at the organizational level [4].

The green transformation of enterprises is a kind of profound organizational change. When an organization sets green transformation as a development goal, employees’ taking charge behavior plays an important role in advancing the goal [10]. As a typical kind of extra-role behavior, employees’ taking charge behavior is a spontaneous and constructive behavior of employees that aims to change and influence organizations by implementing efforts to go beyond their own responsibilities, through more effective ways of working [11]. Research on proactive behavior has indicated the relationship among different concepts, such as extra-role behavior, organizational citizenship behavior, and taking charge behavior. Proactive work behavior includes in-role and extra-role behavior [12]. Extra-role behavior includes organizational citizenship behavior, voice behavior, helping behavior, and taking charge behavior [12,13,14,15,16]. It should be noted that although these concepts have some similarities, they differ significantly in terms of connotations and measurements. Morrison and Phelps (1999) pointed out that employees’ taking charge behavior is remarkably different from organizational citizenship behavior; for example, compared with organizational citizenship behavior that is affiliative-promotive, taking charge behavior is more change-oriented and aimed at procedure improvement, bringing changes and innovative solutions to organizations [13]. A review of the relevant literature showed that the influencing factors of employees’ taking charge behavior consists of two main aspects. The first is an exploration of the intrinsic elements that drive employees’ taking charge behavior from a cognitive and psychological perspective. Dysvik, Kuvaas, and Buch (2016) described the pathways by which employees’ intrinsic perceptions of organizational development can influence employees’ taking charge behavior [11]. Love and Dustin (2014) explored the practical impact of emotional factors, such as psychological collectivism, on employees’ taking charge behavior, starting with the leader–member relationship and other aspects [17]. Second, the external influencing factors on employees’ taking charge behavior are explored in terms of the organizational environment. For example, support from both organizations and leaders facilitate employees’ taking charge behavior [18]. Leadership and allocation systems in the organization can also significantly affect employees’ taking charge behavior [19]. In studies of the influencing factors of employees’ taking charge behavior, leadership has received widespread attention from scholars. As an emerging concept in the field of green management, green transformational leadership, which includes green influence, green motivation, green intellectual stimulation, and green personalized consideration as the main modes of action, contributes significantly to shaping employees’ green vision and motivating them to achieve green goals [4]. Since the concept of green transformational leadership was introduced, it has received considerable attention from scholars in the field of green management [20]. Prior studies have focused on the mechanisms of the effect of transformational leadership on employees’ taking charge behavior [21,22]. However, Robertson (2018) demonstrated that when transformational leadership is introduced into the field of green management and its functions are explored, there are many differences from the original concept of transformational leadership in terms of indicators, influencing mechanisms, and transmission pathways [23].

Prior research has shown that green transformational leadership is positively related to organizational citizenship behavior [7] and organizational citizenship behavior for the environment [8,9]; however, as we mentioned above, employees’ taking charge behavior is different from organizational citizenship behavior in several aspects. Organizational citizenship behavior places more emphasis on affiliative behaviors such as helping, sharing, and cooperating, which is the modest behavior that sustains the status quo [24]. As a new kind of OCB, organizational citizenship behavior for the environment (also called organizational citizenship behavior toward the environment, environmental citizenship behavior) is defined as individual and discretionary behaviors that are not explicitly recognized by the formal work rules and that contribute to the protection of the environment [25], including eco-civic engagements, eco-initiatives, and eco-helping behavior of employees [26], which is still in the domain of OCB. In contrast, taking charge behavior is more targeted at changing the organization [12,26]. It involves risk taking, which means that taking charge behavior is more challenging than other types of extra-role behavior, such as organizational citizenship behavior [27,28,29]. Currently, few studies have revealed the influencing mechanisms and boundary conditions of green transformational leadership on employees’ taking charge behavior. Therefore, it is necessary to further explore the relationship between green transformational leadership and employees’ taking charge behavior in the context of green transformation.

To fill these research gaps, we introduce self-determination theory, which divides the motivations that drive individuals to accomplish their goals into intrinsic and extrinsic motivations [30], and further explains the elements of the external environment that drive employees to accomplish organizational goals [31]. The introduction of self-determination theory facilitates our exploration of the influencing mechanisms of green transformational leadership on employees’ taking charge behavior, allowing our study to integrate the extrinsic motivation provided by the organization and the driving force based on individuals’ intrinsic motivation. Among the intrinsic motivations for employees’ taking charge behavior, personal initiative can reinforce employees’ sense of responsibility and prosocial motivation [32], and self-efficacy [33], which in turn supports employees’ taking charge behavior. Therefore, we adopted personal initiative as a mediating variable in the model for our study. In exploring external motivations for taking charge behavior, the role of green organizational identity cannot be ignored because it contributes to the construction of organizational green goals and promotes the formation of personal initiative, which in turn strengthens the relationship between green transformational leadership and employees’ taking charge behavior [34]. Based on these mechanisms, we introduce green organizational identity as a moderating variable in the model.

We used two-stage questionnaire surveys to collect data from 429 employees in manufacturing enterprises undergoing a profound green transformation and conducted hierarchical multiple regression analysis to test the model. The results indicate that green transformational leadership has a positive impact on employees’ taking charge behavior, and that personal initiative plays a partial mediating role in the above relationship. Furthermore, green organizational identity positively moderates both the relationship between green transformational leadership and personal initiative and the indirect effect of green transformational leadership on employees’ taking charge behavior via personal initiative. Our study makes several contributions to the existing research and expands the theoretical understanding of green transformational leadership. The study incorporates personal initiative and green organizational identity into the influencing mechanisms of green transformational leadership on employees’ taking charge behavior for the first time, which introduces a new perspective on the study of green transformational leadership and enriches research on the boundary conditions of how green transformational leadership influences employee behavior. At the same time, the integration of these factors provides theoretical support for the application of self-determination theory in green management. Our study also provides a comprehensive response to the effect of various individual and organizational factors on employees’ taking charge behavior and the sustainable development of organizations.

## 2. Theoretical Background and Hypothesis Development

### 2.1. Green Transformational Leadership and Employees’ Taking Charge Behavior

Transformational leadership is defined as a leadership structure based on trust and commitment, which positively influences followers’ motivation, identity, and goal achievement by shaping their confidence, self-efficacy and self-esteem [35]. The concept of transformational leadership covers four dimensions: idealized influence, inspirational motivation, intellectual stimulation, and personalized consideration [35,36]. The study of transformational leadership did not initially focus on a specific area, and researchers explored the nature and effects of transformational leadership in a more general context [23]. For example, Bass (1999) explored the factor model of transformational leadership and compared the effects of transformational leadership with those of transactional leadership from a wider perspective [37]. Building on the macrolevel research on transformational leadership, Robertson (2018) recognized the importance of extending transformational leadership to the environmental context; therefore, transformational leadership was extended to the field of green management to develop the concept of green transformational leadership [23]. Most of the current research uses Chen and Chang’s description of green transformational leadership, in terms of motivating subordinates to achieve green goals and inspiring them to achieve environmental performance beyond expected levels [4,38]. The effectiveness of transformational leadership is measured and quantified in broad terms, including a wide range of behaviors that enhance organizational effectiveness. Unlike the scope of research on transformational leadership in general, by refining and focusing on indicators such as employees’ task performance, innovation performance, and other indicators in the area of environmental protection and environmental friendliness, Robertson and Barling (2013) elaborated the positive impact of green transformational leadership on employees’ pro-environmental behavior [39], and used methods such as discriminant validity tests to illustrate the conceptual and measurement differences between green transformational leadership and transformational leadership, broadening the research paradigm on the effects of green transformational leadership [23].

Previous studies have shown that green transformational leadership is positively correlated with organizational citizenship behavior [7] and organizational citizenship behavior for the environment [8,9]. Although organizational citizenship behaviors, such as helping others with workloads and obeying informal organizational norms, might contribute to perpetuating organizational procedures and routines, such uncritical support and compliance may be contrary to the rapid development of companies in the era of change [14,24,28]. Therefore, it is necessary to propose a different conceptualization of extra-role behavior, called taking charge behavior [12], which emphasizes the before-the-fact “assumed responsibility” for the change and future development of organizations, rather than the after-the-fact “assigned responsibility” [28]. Chiaburu and Baker (2006) explored the distinctiveness of taking charge behaviors from organization-directed and individual-directed organizational citizenship behaviors [29]. Compared with organizational citizenship behavior that is affiliative-promotive, taking charge behavior is more challenging-promotive, including adopting improved procedures for the work, changing the way to improve efficiency, or correcting faulty procedures or practices [24,29]. As an important extra-role behavior, employees’ taking charge behavior is defined as the spontaneous and constructive work behavior of organizational members that is designed to change and influence the organization [12]. Employees’ taking charge behavior can effectively enhance the sustainable development of an organization and contribute to the green change of the enterprise [40]. According to Morrison and Phelps (1999), the factors that influence employees’ taking charge behavior are the perceived openness of top management, the perception of organizational change, self-efficacy, and employees’ responsibility [13]. We assume that employees’ perceptions of green transformational leadership can influence taking charge behaviors in several ways. First, green transformational leaders improve employees’ perceptions of organizational openness by changing their values and goals toward green management, which in turn encourages employees to practice taking charge behaviors that go beyond their assigned responsibilities [40]. Green transformational leaders’ environmentally friendly exploration of green development increases employees’ interest in environmental issues, raising their awareness of green organizational change and innovation [41]. Green transformational leaders provide effective support for environmentally friendly behavior, thus reinforcing internal and external motivations for taking charge behavior and fostering environmental self-efficacy among employees [42]. Green transformational leaders help employees understand green work styles and values so that they can foster commitments to environmentally friendly workflows, which develops employees’ interest in taking charge behavior and motivates them to achieve environmental goals [43]. Based on the above discussion, the following hypothesis is proposed:

**Hypothesis** **1** **(H1).** *Green transformational leadership is positively associated with employees’ taking charge behavior.*

### 2.2. The Mediating Role of Personal Initiative

Personal initiative is defined as an inner drive to go beyond the scope of one’s duties and job requirements and to take a more proactive approach to work [44]. According to Frese, Fay, and Hilburger (1997), personal initiative covers the following aspects: (1) consistency with the organization’s mission; (2) goal directed and action oriented; (3) long term; (4) self-starting and proactive; and (5) persistent in the face of barriers and setbacks [44]. Previous research has linked transformational leadership to personal initiative. For example, Herrmann and Felfe (2014) integrated transformational leadership, personal initiative, and creativity into one model and illustrated the positive impact of transformational leadership on personal initiative [45]. Den Hartog and Belschak (2012) examined a combination of transformational leadership, role breadth self-efficacy, and personal initiative to explain the positive effects of transformational leadership on the personal initiative of employees with high self-efficacy [46]. The measures of personal initiative also emphasize responding to difficulties and seizing opportunities [44]. Chen, Shih, and Yeh (2011) developed a two-item scale of personal initiative based on Frese et al.’s work, which includes “do work automatically” and “do the work better and faster than supervisor expected” [47]. The scale used in our study is aligned with the above study. Specifically, we place emphasis on the role of personal initiative as an intrinsic drive rather than on what specific behaviors personal initiative represents. The reasons why we consider personal initiative as the affective factor and intrinsic motivation are as follows. First, the conceptual framework of personal initiative is closely related to affective motivation, such as self-efficacy and work engagement, at which point it is clearer to illustrate the intrinsic driving paths and mediating role of personal initiative in our study [47]. Second, since taking charge behavior is a future-oriented behavior, the antecedents of taking charge behavior are highly relevant to connotations of personal initiative, for instance, mentally preparing for future situations and seizing opportunities [48,49]. This not only helps to elaborate the influencing mechanism more clearly, but also facilitates the use of self-determination theory in this study.

Self-determination theory classifies individuals’ motivation to engage in activities as intrinsic motivation based on interest and extrinsic motivation based on the value of the output of the activity [50]. According to self-determination theory, we can explain the influencing mechanism of green transformational leadership on personal initiative from the following perspectives. First, previous research has shown that setting goals that are long-term and beyond personal responsibility is an important basis for personal initiative [51]. Through green motivation inspiration, green transformational leaders encourage employees to go beyond their personal interests and practice long-term, pro-environmental ways of working that are aligned with organizational goals. Green transformational leaders enhance employees’ green autonomous motivation by motivating them to actively internalize green values [23]. Through green motivation inspiration, leaders can create environmental norms and values in the workplace and allow employees who practice environmentally friendly ways of working to experience a sense of personalized care, which in turn enhances environmental extrinsic motivation and lays the foundation for improving employees’ personal initiatives [51]. When employees encounter workplace obstacles, leaders can enhance their employees’ green innovation competency through green intellectual stimulation and green influence, which in turn improves their ability and persistence to achieve the organization’s environmental goals, thus stimulating their personal initiative for pro-environmental taking charge behavior [52,53]. Therefore, we propose the following hypothesis:

**Hypothesis** **2** **(H2).** *Green transformational leadership is positively related to personal initiative.*

Personal initiative is an important influencing factor of taking charge behavior. Research on intraindividual factors, such as self-efficacy and sense of responsibility, has expanded the perspective on the personal initiative formation process and provided a theoretical basis for studying the influence of personal initiative on taking charge behavior. Personal initiative is reflected in the organization as responding to difficulties and seizing opportunities [54]. Based on Morrison and Phelps’ study [13] on factors that influence taking charge behavior, there are several ways in which personal initiative can influence employees’ taking charge behavior. First, personal initiative increases individual motivation in the face of obstacles and difficulties and increases the likelihood of performing challenging tasks, which enable employees to develop an optimistic view of work and enhance their self-efficacy, thus promoting taking charge behavior [54]. According to Zacher et al.’s study on the emotional utility of personal initiative, changes in personal initiative have an impact on emotional engagement and emotional exhaustion [33]. Changes in personal initiative and emotion can moderate employees’ felt responsibility and stimulate work commitment beyond the call of duty, which in turn influences taking charge behavior [55]. The increase in personal initiative also implies employees’ acceptance of organizational change and openness, such as an environmentally friendly working atmosphere [56]. The perception of organizational change and organizational openness is an important basis for taking charge behavior. Based on the above theories, we propose the following hypothesis:

**Hypothesis** **3** **(H3).** *Personal initiative mediates the positive relationship between green transformational leadership and employees’ taking charge behavior.*

### 2.3. The Moderating Role of Green Organizational Identity

Organizational identity is defined as the process by which an individual’s beliefs about his or her organization are recognized and integrated into his or her personal perception of the organization [57]. According to previous research, the factors that influence organizational identity include organizational characteristics, organizational culture and communication, job support, and motivation [58]. Previous research on the moderating effect of organizational identity focused on the cognitive and psychological processes of employees to explain the role played by organizational identity in the relationship between leadership behaviors and personal initiative [59]. For example, Van Dick, Grojean, and Christ (2006) illustrated the positive impact of organizational identity on employees’ work attitudes from an emotional value perspective by integrating organizational identity, individual work motivation, and taking charge behavior into a model [60]. By quantifying organizational identity, Riketta (2005) developed the idea that leadership styles can make contributions to employees’ organizational identity [61]. Green organizational identity is a concept formed by extending organizational identity to the green domain, which is defined as the meaning and interpretative scheme of environmental protection behavior within an organization that is collectively constructed by organizational members [10]. Based on the above intrinsic association of green organizational identity with green transformational leadership and personal initiative, we infer that green organizational identity may be an important moderating variable in the relationship between green transformational leadership and personal initiative.

Self-determination theory suggests that individuals have an extrinsic motivation to assimilate and integrate external values, which can be progressive in accordance with the degree of employee autonomy. The most direct external motivation is the external regulation formed by employees who recognize the rewards and benefits of the activity. If employees partially recognize the importance of the activity, this is partially autonomous identified regulation. On this basis, employees who integrate the value of the activity as part of their self-worth can form fully autonomous integrated regulation, which has a deeper impact on efficiency and behavior [53]. It is evident that as the autonomy of extrinsic motivation varies, the impact factors, such as green transformational leadership, on personal initiative can also vary. According to self-determination theory, green organizational identity can moderate the relationship between green transformational leadership and personal initiative in several ways. First, green organizational identity allows employees to more effectively identify the recognition and motivation of their leaders for their contributions [62,63], leading to a stronger perception of the instrumental value of green behaviors and enriching the external regulation that enhances environmental initiatives, which in turn enhances the impact of green transformational leadership on personal initiative. Second, a high level of green organizational identity helps employees feel less controlled by their leaders and creates a greater sense of willingness and autonomous choice in the execution of their tasks [52]. As a result, the external regulation of environmentally friendly ways of working has the potential to be transformed into autonomous external motivation, thus enhancing the influence of green transformational leadership on personal initiative from external instrumental values to personal willingness. Third, by building a high level of green organizational identity, green transformational leaders integrate green emotional ties into their management behavior [64], which facilitates employees’ development of confidence and values in the organization’s green initiatives [43] and constructs integrated regulation for environmentally friendly ways of working. Green organizational identity, therefore, helps to construct high-level extrinsic motivation, which in turn enhances the influence of green transformational leadership on personal initiative. Therefore, we propose the following hypothesis:

**Hypothesis** **4** **(H4).** *Green organizational identity positively moderates the relationship between green transformational leadership and personal initiative, which is stronger when green organizational identity is higher than when it is lower.*

**Figure 1:**
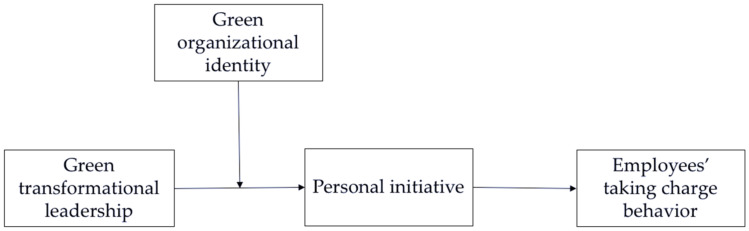
Proposed model.

By combining hypotheses 1, 2, 3, and 4, we further propose that green organizational identity can moderate the mediating role of personal initiative in the effect of green transformational leadership on employees’ taking charge behavior, which is a moderated mediation model. Specifically, when the degree of green organizational identity is higher, employees are better able to understand green transformational leadership behaviors and to form green values and a sense of responsibility, so that they are more effectively motivated to take personal initiative and engage in taking charge behavior. Consequently, the indirect effect of green transformational leadership on employees’ taking charge behavior through personal initiative will be more positive. Therefore, the following hypothesis is proposed, and Figure 1 shows the proposed model.

**Hypothesis** **5** **(H5).** *Green organizational identity positively moderates the mediating effect of personal initiative on the relationship between green transformational leadership and employees’ taking charge behavior, such that the indirect effect is stronger when green organizational identity is high.*

## 3. Method

### 3.1. Sample and Procedure

In the context of carbon neutrality, manufacturing enterprises are currently undergoing profound green changes. These green management activities, which aim to improve green innovation and seek to conserve resources, are not only an important basis for carbon peaking but also provide convenient quantitative indicators for our study. Therefore, the data were collected in the manufacturing industry. The questionnaires used for the study were collected from 26 manufacturing enterprises in eastern China. Data were collected in two stages at 4-week intervals to control for common method deviation. In the first wave (Time 1), employees were required to provide demographic information, including gender, age, organizational tenure, and education, and evaluate their perceptions of green transformational leadership and their own personal initiative. In the second wave (Time 2), evaluations of employees’ ratings of their taking charge behavior and green organizational identity were collected. A total of 590 questionnaires were distributed in our study. After screening out missing and invalid samples, there were 429 complete and valid questionnaires, with a valid response rate of 72.71%. Demographically, 299 respondents were male (69.46%). The average age of employees is 32.71 years (SD = 6.66). In terms of education, 400 respondents had junior college or bachelor’s degrees (93.2%). The average organizational tenure of respondents is 3.07 years (SD = 1.38).

### 3.2. Measures

In designing the questionnaire used to collect the relevant constructs, we followed the back-translation method to translate the questionnaire [65]. We also adapted the questionnaire to the specific cultural context of China. Employees rated all items on a 5-point Likert scale in ascending order from “strongly disagree” to “strongly agree”.

Green transformational leadership. Green transformational leadership was evaluated using the 6-item green transformational leadership scale [4]. Example items include “My supervisor of the green product development project inspires the project members with environmental plans” and “My supervisor of the green product development project provides a clear environmental vision for the project members to follow”. The Cronbach’s alpha for this measure was 0.86.

Personal initiative. Employees evaluated their personal initiative using a 7-item personal initiative scale [44]. Example items include “I actively attack problems” and “Whenever there is a chance to get actively involved, I take it”. The Cronbach’s alpha for this measure was 0.92.

Green organizational identity. Employees evaluated their green organizational identity using a 6-item scale developed by Chen [10]. Example items include “The company’s top managers, middle managers, and employees have a strong sense of the company’s history about environmental management and protection” and “The company’s top managers, middle managers, and employees have a sense of pride in the company’s environmental goals and missions”. The Cronbach’s alpha for this measure was 0.94.

Taking charge behavior. Employees evaluated their taking charge behavior using a 3-item scale [40]. Example items include “How frequently do you try to institute new work methods that are more effective?” and “How frequently do you try to bring about improved procedures in your workplace?”. The Cronbach’s alpha for this measure was 0.90.

## 4. Results

### 4.1. Common Method Bias Test

Considering the homogeneity of the data sources, the data of this study may be subject to the problem of common method bias. To test this issue, an exploratory factor analysis was conducted with Harman’s one-factor test using SPSS 26 (IBM, New York, NY, USA) for all indicators. The first factor could explain only 21.34% of the variance, indicating that CMV was not a serious problem in our survey.

### 4.2. Descriptive Statistics

Table 1 presents the means, standard deviations, and correlations of all variables in our study. As indicated in Table 1, green transformational leadership is positively correlated with personal initiative (*r* = 0.57, *p* < 0.01) and taking charge behavior (*r* = 0.61, *p* < 0.01). Personal initiative is positively correlated with taking charge behavior (*r* = 0.64, *p* < 0.01). Thus, the hypotheses are preliminarily verified.

### 4.3. Confirmatory Factor Analyses

We conducted confirmatory factor analyses (CFAs) to verify the validity of these constructs in our study by using Mplus 8 (Muthén & Muthén, Los Angeles, CA, USA). As reported in Table 2, the CFA results indicate that our hypothesized four-factor model (green transformational leadership, personal initiative, green organizational identity, taking charge behavior) provided a better fit to the data (CFI = 0.906; TLI = 0.913; RMSEA = 0.095; SRMR = 0.049) than other models. The above results revealed that the model we proposed had the best validity.

### 4.4. Hypothesis Testing

Before hypothesis testing, we used the variance inflation factor (VIF) method to test for multicollinearity. The VIFs for green transformational leadership (1.72), personal initiative (1.71), and green organizational identity (1.74) were below 5, demonstrating that multicollinearity was not a serious problem in this study.

We conducted hierarchical regression analysis using the SPSS PROCESS macro developed by Hayes and Scharkow [66] to test the hypotheses. The results of hierarchical regressions are presented in Table 3. Model 1 regressed the effect of control variables on personal initiative (PI). Model 2 regressed the effect of green transformational leadership (GTL) and control variables on PI simultaneously. Model 3 regressed the effect of GTL, green organizational identity (GOI), control variables and the interaction (GTL*GOI) on PI. Model 4 regressed the effect of control variables on employees’ taking charge behavior (TCB). Model 5 regressed the effect of GTL and control variables on TCB simultaneously. Model 6 regressed the effect of GTL, PI, and control variables on TCB.

The relationship between green transformational leadership and taking charge behavior. Hypothesis 1 posited a positive relationship between green transformational leadership and employees’ taking charge behavior. To test this hypothesis, control variables were first regressed on taking charge behavior. Then, green transformational leadership and control variables were regressed on taking charge behavior. The results showed that the effect of green transformational leadership on taking charge behavior was 0.69 (*p* < 0.01) (Table 3, Model 2).

The mediating effect of personal initiative. According to Baron and Kenny (1986) [67], the mediating effect must satisfy the following conditions: (1) the independent variable is significantly related to the dependent variable; (2) the independent variable is significantly associated with the mediator; and (3) the mediator is significantly related to the dependent variable after the independent variable is controlled and the effect of the independent variable on the dependent variable becomes weak (partial mediation) or insignificant (full mediation). Given that H1 is supported, the first condition is satisfied. In Model 2, there is a significant relationship between green transformational leadership and personal initiative (*b* = 0.69, *p* < 0.01) when gender, age, education, and organizational tenure are controlled. Therefore, the second condition is satisfied, and H2 is supported. In Model 6, after controlling for green transformational leadership, the coefficient of personal initiative on taking charge behavior is significant (*b* = 0.54, *p* < 0.01). Furthermore, the relationship between green transformational leadership and taking charge behavior becomes weaker (from *b* = 0.68, *p* < 0.01 in Model 5 to *b* = 0.31, *p* < 0.01 in Model 6). Therefore, the third condition is also satisfied. Combining these conditions, personal initiative partially mediates the relationship between green transformational leadership and taking charge behavior, thus supporting Hypothesis 3. Table 4 shows the direct effect of green transformational leadership on taking charge behavior that excludes the indirect effect of personal initiative. In addition, we employed a bootstrapping test to analyze the mediating effect of personal initiative, which takes the indirect effect into consideration [68,69]. With 5000 bootstrapping tests, the result shows that the indirect effect of green transformational leadership on taking charge behavior via personal initiative is 0.37 (95% CI = [0.29, 0.44]).

**Figure 2:**
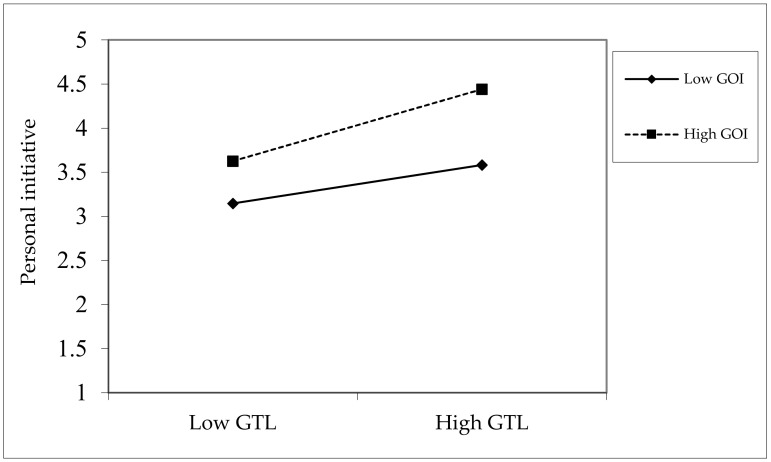
The moderating effect of GOI on GTL and PI.

The moderating effect of green organizational identity. Hypothesis 4 predicts that the relationship between green transformational leadership and employees’ personal initiative will be moderated by green organizational identity. As shown in Table 3, the interaction term (GTL*GOI) on personal initiative is positively significant (*b* = 0.17, *p* < 0.01; Model 3); thus, H4 is supported. To further show the moderating effect of green organizational identity, we plot the effect of green transformational leadership on personal initiative, according to different levels of green organizational identity (high vs. low). As depicted in Figure 2, green transformational leadership is more strongly related to personal initiative when green organizational identity is higher rather than lower, which further verifies Hypothesis 4.

The moderated mediation effect test. We adopted 5000 bootstrapping tests to analyze the moderated mediation model. As shown in Table 5, under the 95% confidence interval, the indirect effects of green transformational leadership on taking charge behavior through personal initiative are significant in the case of both low levels of green organizational identity (mean minus one standard deviation) and high levels of green organizational identity (mean plus one standard deviation). As shown in Table 6, The moderated mediation index is 0.09, and the 95% confidence interval is [0.01, 0.16]. The above results reveal that green organizational identity positively moderates the indirect effect of green transformational leadership on taking charge behavior via personal initiative. Therefore, Hypothesis 5 is supported.

## 5. Discussion

### 5.1. Theoretical Implications

This study expands on the current literature on green transformational leadership and employees’ taking charge behavior and has several theoretical implications. First, the study broadens the research perspective on green transformational leadership by revealing the positive direct and indirect impact of green transformational leadership on employees’ taking charge behavior. Many scholars have explored the impact of green transformational leadership on other outcome variables, such as employees’ green behavior and employees’ green creativity [41]. However, we know little about the mechanisms and pathways by which green transformational leadership influences employees to go beyond their own responsibilities and take the initiative to work constructively toward organizational green goals. This paper extends the research on the utility of green transformational leadership to employees’ taking charge behavior and illustrates the mechanisms by which green transformational leadership influences taking charge behavior.

Second, this paper introduces new mediating and moderating variables to the study of green transformational leadership, namely, personal initiative and green organizational identity. By introducing self-determination theory to our study, we explain how green transformational leadership has a long-term and sustainable effect on personal initiative, in terms of intrinsic and extrinsic motivation, and illustrate the role of personal initiative in motivating taking charge behavior. By illustrating the mediating role of personal initiative, our study not only supports the previous claim that green transformational leadership can enhance an individual’s intrinsic motivation to protect the environment [5], but also enriches the study of the influencing mechanism of green transformational leadership from the perspective of motivation. At the same time, this paper illustrates the moderating effect of green organizational identity on the relationship between green transformational leadership and taking charge behavior based on self-determination theory, which presents different levels of extrinsic motivation for personal initiative. At different levels of green organizational identity, the impact of green transformational leadership on personal initiative and employees’ taking charge behavior may produce a large efficiency gap due to the different levels of internalization of organizational green values by employees.

This paper also broadens the boundary conditions of self-determination theory. By introducing self-determination theory into this study, we unify green organizational identity and personal initiative into one model, which provides an example of the green management aspects of self-determination theory and enriches the direction of the application of self-determination theory [70]. Moreover, the moderating effect of green organizational identity at different levels supports the significance of Ryan and Deci’s subdivision of external motivation by individual autonomy [53]. This study also provides a reference for the application of self-determination theory in the field of green management and promotes the development of self-determination theory.

### 5.2. Practical Implications

As interest in the concept of carbon neutrality continues to grow globally, research on pro-environmental behavior is gradually advancing [71]. In the area of green management, our research has some practical implications. First, our research shows that green transformational leadership has a positive impact on employees’ taking charge behavior. Therefore, organizations should encourage leaders to practice green behaviors and establish an environmentally friendly role model for employees within the organization through their own influence, by participating in decision-making and taking relevant responsibility. At the same time, through the establishment of green performance indicators and environmental training, leaders can foster employees’ internal and external motivation for taking charge behavior and motivate them to conserve resources and protect the environment. In addition, based on the heterogeneity of each employee’s individual needs, leaders should implement personalized care and provide relevant individualized intellectual stimulation and training so that each employee has the ability and willingness to engage in taking charge behavior as much as possible.

Second, green transformational leaders need to be aware of the role of personal initiative in motivating taking charge behavior and enhancing employees’ initiative and innovation when faced with green organizational change. To this end, leaders can emphasize the significance of environmentally friendly ways of working to enhance employees’ felt responsibility and implement material and moral rewards for employees who achieve better green goals. At the same time, leaders need to pay attention to the emotional factors of employees that minimize their sense of being in control in the process of green organizational change so that employees can actively seek and build internal and external motivation to practice taking charge behavior.

Green organizational identity profoundly affects the effectiveness of green transformational leadership. Leaders should take the initiative to integrate green management concepts into the organizational identity framework and enhance employees’ understanding of environmental strategies. At the same time, leaders should give meaning to green organizational change and integrate emotional connections into the green management model so that employees can more effectively identify and perceive green transformational leadership and thus take more responsibility for the environment.

### 5.3. Limitations and Future Research

First, green transformational leadership is a concept that can be refined into multiple dimensions, such as green influence and green intellectual stimulation. This study only examines green transformational leadership as a whole. In the future, green transformational leadership can be refined into multiple dimensions to more precisely elaborate its effect on employees’ taking charge behavior. Additionally, further studies can also explore how other types of pro-environmental leadership, such as sustainable leadership, influence taking charge behavior.

Second, although we used a two-wave questionnaire for the study, the data were measured from self-reports by employees. Due to this limitation, the data results may be subjective. Future research could collect data from multiple sources to reduce common method bias.

Third, green organizational identity was examined at the individual level. In the future, green organizational identity can also be measured as an organizational-level variable to more comprehensively assess the moderating effect of green organizational identity.

## 6. Conclusions

Based on self-determination theory, this study examined the influencing mechanisms and boundary conditions of the effect of green transformational leadership on employees’ taking charge behavior. The empirical evidences revealed that green transformational leadership is positively associated with employees’ taking charge behavior, and personal initiative mediates the positive relationship between green transformational leadership and employees’ taking charge behavior. Furthermore, the study also confirmed that green organizational identity positively moderates the relationship between green transformational leadership and personal initiative, and the indirect relationship between green transformational leadership and employees’ taking charge behavior through personal initiative.

## Figures and Tables

**Table 1:** Means, standard deviations and interrelations of variables.

Variable	Mean	SD	1	2	3	4	5	6
1. Age	32.71	6.66						
2. OT	3.07	1.38	0.58 **					
3. Edu	0.87	0.50	−0.03 *	−0.14 *				
4. GTL	4.18	0.68	0.06	0.11 **	−0.06			
5. GOI	4.15	0.82	0.14 **	0.13 *	−0.04	0.58 **		
6. PI	3.90	0.82	0.02	0.04	−0.09	0.57 **	0.58 **	
7. TCB	3.95	0.76	0.09	0.11 *	−0.08	0.61 **	0.73 **	0.72 **

*N* = 429. OT represents organizational tenure; Edu represents education; GTL represents green transformational leadership; GOI represents green organizational identity; PI represents personal initiative; TCB represents taking charge behavior. * *p* < 0.05; ** *p* < 0.01.

**Table 2:** Confirmatory factor analysis.

Factor Structure	χ^2^	Δχ^2^	CFI	TLI	RMSEA	SRMR
Four-factor model (GTL; PI; GOI; TCB)	894.006		0.906	0.913	0.095	0.049
Three-factor model (combining GTL and GOI together)	1537.017	804.966 **	0.834	0.845	0.126	0.078
Three-factor model (combining GTL and PI together)	1574.472	767.511 **	0.83	0.83	0.127	0.085
Two-factor model (combining GTL, PI,OI together)	2341.983	925.627 **	0.772	0.748	0.147	0.094
One-factor model (combining all items into one factor)	3267.61	6236.205 **	0.672	0.639	0.176	0.094

Note. CFI, Comparative Fit Index; TLI, Tucker–Lewis Index; RMSEA, root mean squared error of approximation; SRMR, standardized root mean square residual. ** p < 0.01.

**Table 3:** Results of hierarchical multiple regression.

	Personal Initiative			Taking Charge Behavior		
	M1	M2	M3	M4	M5	M6
CV						
Gender	−0.07	−0.09	−0.06	0.05	0.03	0.08
Age	−0.01	−0.01	−0.01	0.01	0.01	0.01
OT	0.03	−0.01	−0.01	0.04	0.01	0.01
Edu	−0.17	−0.12	−0.13	−0.07	−0.03	0.04
IV						
GTL		0.69 **	0.46 **		0.68 **	0.31 **
Mediator						
PI						0.54 **
Moderator						
GOI			0.41 **			
Interaction						
GTL*GOI			0.17 **			
R^2^	0.01	0.32	0.44	0.02	0.38	0.59
F	1.19	42.14 **	48.02 **	1.82	51.27 **	102.97 **

*N* = 429. CV represents control variable; IV represents independent variable; OT represents organizational tenure; Edu represents education; GTL represents green transformational leadership; GOI represents green organizational identity; PI represents personal initiative; TCB represents taking charge behavior. ** p < 0.01.

**Table 4:** Regression analysis of the mediating effect.

Effect	B	SE	LLCI	ULCI
Direct effect of X on Y	0.31 **	0.04	0.23	0.40
Indirect effect of X on Y	0.37 **	0.04	0.29	0.44
Total effect of X on Y	0.68 **	0.04	0.59	0.76

** *p* < 0.01.

**Table 5:** Conditional indirect effect at specific values of green organizational identity.

Moderator	Effect	SE	LLCI	ULCI
Low	0.17	0.04	0.09	0.26
Mean	0.24	0.04	0.17	0.33
High	0.32	0.06	0.21	0.44

**Table 6:** Index of moderated mediation.

Outcome	Index	SE	LLCI	ULCI
TCB	0.09	0.04	0.01	0.16

## Data Availability

The data presented in this study are available on request from the corresponding author. The data are not publicly available due to respondents’ privacy.
